# Inter-Vendor Reproducibility of Myelin Water Imaging Using a 3D Gradient and Spin Echo Sequence

**DOI:** 10.3389/fnins.2018.00854

**Published:** 2018-11-21

**Authors:** Lisa Eunyoung Lee, Emil Ljungberg, Dongmyung Shin, Chase R. Figley, Irene M. Vavasour, Alexander Rauscher, Julien Cohen-Adad, David K. B. Li, Anthony L. Traboulsee, Alex L. MacKay, Jongho Lee, Shannon H. Kolind

**Affiliations:** ^1^Department of Medicine, The University of British Columbia, Vancouver, BC, Canada; ^2^Department of Neuroimaging, Institute of Psychiatry, Psychology & Neuroscience, King’s College London, London, United Kingdom; ^3^Department of Electrical and Computer Engineering, Seoul National University, Seoul, South Korea; ^4^Department of Radiology, University of Manitoba, Winnipeg, MB, Canada; ^5^Department of Radiology, The University of British Columbia, Vancouver, BC, Canada; ^6^Department of Pediatrics, The University of British Columbia, Vancouver, BC, Canada; ^7^Department of Physics and Astronomy, The University of British Columbia, Vancouver, BC, Canada; ^8^NeuroPoly Lab, Institute of Biomedical Engineering, Polytechnique Montreal, Montreal, QC, Canada; ^9^Functional Neuroimaging Unit, CRIUGM, Université de Montréal, Montreal, QC, Canada

**Keywords:** myelin water imaging, GRASE, reproducibility, quantitative imaging, multi-site, multi-vendor, magnetic resonance imaging

## Abstract

Myelin water imaging can be achieved using multicomponent T_2_ relaxation analysis to quantify *in vivo* measurement of myelin content, termed the myelin water fraction (MWF). Therefore, myelin water imaging can be a valuable tool to better understand the underlying white matter pathology in demyelinating diseases, such as multiple sclerosis. To apply myelin water imaging in multisite studies and clinical applications, it must be acquired in a clinically feasible scan time (less than 15 min) and be reproducible across sites and scanner vendors. Here, we assessed the reproducibility of MWF measurements in regional and global white matter in 10 healthy human brains across two sites with two different 3 T magnetic resonance imaging scanner vendors (Philips and Siemens), using a 32-echo gradient and spin echo (GRASE) sequence. A strong correlation was found between the MWF measurements in the global white matter (Pearson’s *r* = 0.91; *p* < 0.001) for all participants across the two sites. The mean intersite MWF coefficient of variation across participants was 2.77% in the global white matter and ranged from 4.47% (splenium of the corpus callosum) to 17.89% (genu of the corpus callosum) in white matter regions of interest. Bland-Altman analysis showed a good agreement in MWF measurements between the two sites with small bias of 0.002. Overall, MWF estimates were in good agreement across the two sites and scanner vendors. Our findings support the use of quantitative multi-echo T_2_ relaxation metrics, such as the MWF, in multicenter studies and clinical trials to gain deeper understanding about the pathological processes resulting from the underlying disease progression in neurodegenerative diseases.

## Introduction

Quantitative measurement of *in vivo* multicomponent T_2_ relaxation in the central nervous system (CNS) can provide information about pathophysiology based on different water environments in tissues. Myelin is a fatty insulating substance that envelops the axons in the CNS and plays a fundamental role in enabling saltatory conduction and directly supplying energy to axons (Norton and Cammer, 1984; Lee et al., 2012). Quantitative *in vivo* imaging of myelin, using magnetic resonance imaging (MRI), can enhance our understanding about the pathological processes in demyelinating diseases, such as multiple sclerosis (MS), as well as improve the clinical diagnosis, prognosis and disease management process (Laule et al., 2004; Khaleeli et al., 2007; Oh et al., 2007; Kolind et al., 2012, 2015; MacKay and Laule, 2016).

Myelin water imaging (MWI) can be achieved using multi-component T_2_ relaxation analysis to quantify the MR signals from different water compartments within a voxel (MacKay et al., 1994, 2006; Whittall et al., 1997). Healthy tissue in the CNS typically contains cerebrospinal fluid (long T_2_ component of >2000 ms), intra- and extracellular water (intermediate T_2_ component of ∼70 ms) and myelin water in between myelin bilayers (short T_2_ component of ∼15 ms) (MacKay et al., 1994; Whittall et al., 1997). The myelin water fraction (MWF), the ratio of the short T_2_ component (myelin water) to the total T_2_ distribution, shown as the voxel values in MWI, has been used as an *in vivo* marker of myelin content in the CNS (MacKay et al., 1994, 2006; Whittall et al., 1997). MWF has been shown to strongly correlate with histological measures using myelin-specific staining in rats (Webb et al., 2003; Odrobina et al., 2005; Pun et al., 2005), guinea pigs (Gareau et al., 1999, 2000) and postmortem human brains (Moore et al., 2000; Laule et al., 2006). MWI has been widely used to study white matter (WM) abnormalities in MS (Vavasour et al., 2009; Laule et al., 2010; Kolind et al., 2015), schizophrenia (Flynn et al., 2003), phenylketonuria (Sirrs et al., 2007) and traumatic brain injury (Wright et al., 2016).

To effectively apply MWI in multicenter studies and clinically, MWF measurements must be reproducible across sites and scanner vendors. Recently, a combined gradient and spin echo (GRASE) sequence was adopted for MWI, reducing the acquisition time to less than 15 min for full cerebral coverage (20 slices at 5 mm thickness) (Prasloski et al., 2012b).

Previously, Meyers et al. (2013) assessed the reproducibility of MWI with partial brain coverage (7 slices at 5 mm thickness, acquisition time = 18.5 min) in five healthy participants across six sites using a 3D spin echo sequence acquired on 3 T Philips scanners. They demonstrated a good reproducibility of the MWF in the global white matter (WM; intersite coefficient of variation (COV) = 4.68%) (Meyers et al., 2013). The present study follows the methodology of Meyers et al. (2013) but improves on the acquisition technique by using the recently developed rapid whole cerebrum GRASE MWI sequence (Prasloski et al., 2012b) and including 3 T scanners from different vendors. In this study, we assess the reproducibility of MWF measurements in regional and global WM across two sites with different scanner vendors using a 3D GRASE sequence. Demonstrating reproducibility across scanner vendors with a rapid whole brain acquisition technique will bring MWI one step closer to routine use for multisite studies and clinical applications.

## Materials and Methods

### Participant Information

Ten healthy participants (six males and four females; mean age 36.5 years, range 21–53 years) were scanned across two sites with different 3 T MRI scanner vendors. The mean time between the two scans was 26 days (range 5–62 days). All participants had no previously known neurological disorders or brain abnormalities. The study was approved by the Research Ethics Boards at both institutions and all participants provided written informed consent prior to participation.

### Data Acquisition

MRI data were acquired from each participant on a Siemens Magnetom Trio 3 T (Siemens Medical Solutions, Erlangen, Germany) with 32-channel head coil at Seoul National University, Republic of Korea (site 1) and a Philips Achieva 3 T (Philips Medical Systems, Best, The Netherlands) with an 8-channel head coil at the University of British Columbia, Canada (site 2). T_1_-weighted anatomical images were acquired at each site using a whole-brain 3D magnetization-prepared rapid gradient-echo (MP-RAGE) sequence to facilitate automated tissue segmentation and spatial normalization. MWI data were acquired using a whole-brain, multi-echo 3D GRASE sequence (Prasloski et al., 2012b). We were not able to exactly match acquisition parameters at each site due to differences in sequence implementation between the two sites and vendors. Therefore, we chose to use protocols that were standard practice for each site (i.e., that were the most commonly used for ongoing research studies or clinical examinations within that center and which would be representative sample for a future multicenter study), which were as follows:

• Site 1 GRASE: 32 echoes, TE = 10, 20, 30, … 320 ms, TR = 1000 ms, 5 mm slice thickness, 30 slices, slice partial Fourier factor = 5/8, acquisition time = 12 min, 1.5 × 1.5 mm^2^ in-plane resolution.
• Site 1 MP-RAGE: TR = 2400 ms, TE = 2.12 ms, TI = 1000 ms, 1 × 1× 1 mm^3^, α = 8°.
• Site 2 GRASE: 32 echoes, TE = 10, 20, 30, … 320 ms, TR = 1000 ms, 5 mm slice thickness, 20 slices (40 slices reconstructed at 2.5 mm slice thickness), SENSE factor of 2, acquisition time = 14 min, 1 × 1 mm^2^ in-plane resolution.
• Site 2 MP-RAGE: TR = 3000 ms, TE = 3.00 ms, TI = 820 ms, 1 × 1 × 1.6 mm^3^, α = 8°.

### T_2_ Decay Curve Analysis

The 32-echo GRASE sequence produced a T_2_ decay curve in each voxel, which was analyzed using a regularized non-negative least squares (NNLS) algorithm with stimulated echo correction (Whittall and MacKay, 1989; Prasloski et al., 2012a) to obtain the T_2_ distribution (*T*_2_ = 0.015–2 s) for each voxel. The stimulated echo correction adjusts for errors in the refocusing flip angle due to B_1_ inhomogeneity. The extended phase graph algorithm (Prasloski et al., 2012a) was used to calculate a theoretical T_2_ decay curve for non-ideal refocusing pulse flip angle. It then estimated the true refocusing flip angle by comparing theoretical decay curves with eight potential refocusing pulse flip angles linearly spaced from 50 to 180 degrees, to the experimental decay. Using the optimal refocusing pulse flip angle, a T_2_ distribution (40 logarithmically spaced *T*_2_ values from 0.015–2 s) was obtained using NNLS for each voxel. The short T_2_ component attributed to myelin water was identified as 15–40 ms. T_2_ analysis was performed using in-house software code (MATLAB R2013b, The Mathworks, Inc.) developed at the University of British Columbia.

### Global White Matter and Regions of Interest Analyses

The MP-RAGE image was linearly registered and transformed to the first echo of the GRASE data from the same site using FMRIB’s Linear Image Registration Tool (FLIRT) (Jenkinson et al., 2002; Smith et al., 2004). Second, the transformed MP-RAGE image was registered to the MNI-152 2 mm template using a non-linear registration process implemented in FMRIB’s Non-Linear Image Registration Tool (FNIRT) (Smith et al., 2004; Andersson et al., 2007) to obtain the non-linear warp-field between GRASE and MNI-152 space.

**FIGURE 1:**
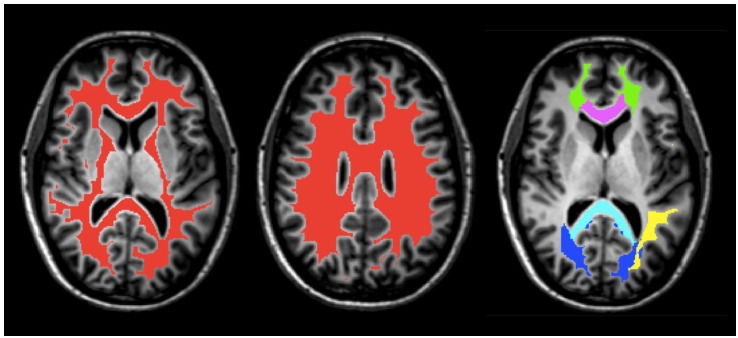
An example of masks of the global WM (red) and ROI, including the splenium (light blue) and genu (purple) of the corpus callosum, superior longitudinal fasciculus (yellow), minor forceps (green), and major forceps (dark blue) on axial T_1_-weighted anatomical image of Participant 1.

Five white matter regions of interest (ROI), including the genu and splenium of the corpus callosum, major and minor forceps, and superior longitudinal fasciculus were obtained from JHU DTI-based white-matter atlases in MNI-152 space (Figure 1) and transformed to GRASE space using the inverse of the previously obtained non-linear warp-field (Wakana et al., 2007; Hua et al., 2008). All ROI were then multiplied by the global WM mask, thresholded and binarized to generate more conservative ROI masks. They were further manually edited when necessary to remove non-WM voxels and to ensure same coverage between the two scans for all participants.

A global WM mask was obtained from the MP-RAGE using FMRIB’s Automated Segmentation Tool (FAST; Figure 1) and subsequently transformed to the native space of the GRASE data using the linear transformation obtained from the first step in the registration process described above (Zhang et al., 2001; Smith et al., 2004). The WM mask was then thresholded, eroded, binarized, and edited when necessary to ensure that non-WM voxels were removed and that the coverage was the same for all participants.

### Statistical Analysis

To assess reproducibility, Pearson’s correlation coefficient (*r*) was calculated using the mean MWF from the global WM of each participant at site 1 and site 2. A *p*-value was calculated from the Pearson’s *r* to determine if the correlation was significant. Statistical significance for all comparisons were defined as *p* < 0.05. A paired *t*-test was performed to determine if there was a difference in mean MWF between two sites. In addition, an equivalence test, two one-sided test (TOST), was performed to determine whether mean MWF between two sites were statistically equivalent. TOST prevents potential misinterpretation of non-significant *p*-values obtained from the paired *t*-test as the absence of a practically important effect. The 95% confidence interval for the estimated difference between the sites is also used to indicate the smallest difference that would have been detectable. The COV was calculated by dividing the standard deviation by the mean, of the two sites, for each ROI and global WM per participant. The COV is biased to lower values when the number of points (*n*) used to calculate COV is small. To correct for this, the sample COV was multiplied by [1 + 1/(4^∗^*n*)] as suggested by Meyers et al. (2013). Here, *n* = 2 so the sample COV was multiplied by 1.13. The corrected COVs were averaged across all participants. Finally, Bland-Altman analysis was used to assess the agreement in MWF between the two sites. All statistical analyses were performed using the R software package.

## Results

**FIGURE 2:**
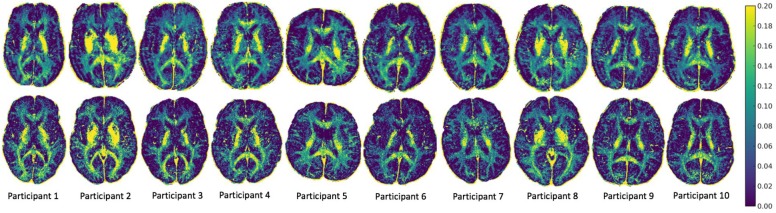
MWF maps from 10 healthy participants from site 1 (top) and site 2 (bottom).

MWF maps of 10 healthy participants from the two sites are illustrated in Figure 2. Tables 1 and 2 display the mean intersite MWF COVs in the global WM and ROI for each participant. The mean intersite MWF COV averaged across the participants was low (2.77%, range 0.03–8.00%). The mean intersite MWF COV was the highest in the genu (17.89%) and lowest in the splenium (4.47%) of the corpus callosum (Table 2).

**Table 1:** Intersite MWF COVs in the global WM for each participant.

Participant	MWF COV (%)
1	0.03
2	1.01
3	1.03
4	1.04
5	1.98
6	2.51
7	2.53
8	3.46
9	6.11
10	8.00
Mean	2.77

**Table 2:** Summary of mean intersite MWF COVs in the global WM and ROI averaged across 10 participants.

ROI	Mean intersite MWF COV (%)
Splenium of the corpus callosum	4.47
Superior longitudinal fasciculus	10.86
Minor forceps	12.71
Major forceps	6.63
Genu of the corpus callosum	17.89
Global white matter	2.77

**FIGURE 3:**
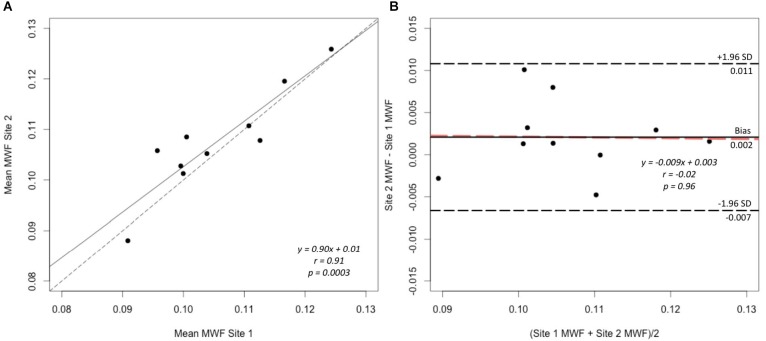
Correlation plot of mean MWF **(A)** and Bland–Altman plot comparing MWF measurements **(B)** between site 1 and site 2 in the global WM mask across 10 healthy participants. The solid line represents the slope of the MWF data and the dashed line represents y = x, which indicates 1:1 agreement, on the correlation plot **(A)**. The black solid line represents the average difference (bias), the black dashed lines indicate the limits of agreement (±1.96 standard deviation) and the orange dashed line indicates the linear fit to the data point **(B)**.

**FIGURE 4:**
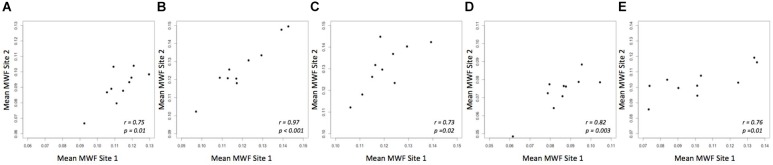
Mean MWF measurements between site 1 and site 2 in genu **(A)** and splenium of the corpus callosum **(B)**, major forceps **(C)**, minor forceps **(D)** and superior longitudinal fasciculus **(E)** across 10 healthy participants.

A strong correlation was observed between the mean MWF in the global WM at each site (Pearson’s *r* = 0.91, *p* < 0.001) (Figure 3). The estimated difference between sites for the global WM was −0.002. A paired *t*-test was not statistically significant (*t* = −1.480, *p* = 0.173) with a 95% confidence interval of −0.005–0.001 indicating that the smallest detectable difference would have been 0.003. Based on the equivalence test, the observed estimated effect of −0.002 was statistically equivalent to zero (*p* = 0.03). This test was based on equivalence bounds of −0.005 and 0.005, an alpha of 0.05 and the 90% confidence interval of −0.0047–0.0005. The variation in the MWF measurements in the global WM between the two sites was not associated with a longer time between the two scans (mean time between the scans = 26 days, range 5–62 days; *r* = 0.14, *p* = 0.70). The mean MWF for the individual ROI are shown in Figure 4 as a scatter plot. There was a high correlation between the mean MWF in the genu (*r* = 0.75, *p* = 0.01) and splenium (*r* = 0.97, *p* < 0.001) of the corpus callosum, major forceps (*r* = 0.73, *p* = 0.02), minor forceps (*r* = 0.82, *p* = 0.003) and superior longitudinal fasciculus (*r* = 0.76, *p* = 0.01) at each site (Figure 4).

Bland-Altman analysis showed good agreement in the MWF measurements in the global WM between the two sites (Figure 3). There was a very small bias of 0.002 with the upper limit of agreement (+1.96 standard deviation) of 0.011 and lower limit of agreement (−1.96 standard deviation) of −0.007. The data points were distributed evenly around the bias, as indicated by the non-significant slope (*p* = 0.96) (Figure 3).

## Discussion

In this study, we extended previous findings from a multisite and single-vendor study of MWF reproducibility (Meyers et al., 2013). We assessed the reproducibility of MWF at two different sites using MR scanners of different vendors (Siemens and Philips) and found good intersite global WM mean MWF reproducibility indicated by a low COV (mean 2.77%, range 0.03–8.00%), high Pearson’s correlation coefficient (*r* = 0.91, *p* < 0.001) and very small bias (mean bias = 0.002). The COV remained low when comparing the 25th (6.04%) and 75th percentile (4.27%) of MWF in global WM across all participants between sites. In comparison, Meyers et al. (2013) found an intersite mean MWF COV (mean 4.68%, range 2.86–8.14%) in the global WM for five healthy participants scanned at six sites using the same MR scanner vendor. Meyers et al. (2013) also showed wider bands between the mean bias and ± 1.96 standard deviation (−0.014–0.016) on Bland–Altman plot, which indicates a greater uncertainty compared to our study (−0.007–0.011). Assuming the true mean difference is the worst case of −0.005, the lower boundary of the 95% confidence interval from a paired *t*-test, this gives a ±4.74% change on average based on our MWF data for global WM. Based on our equivalence bounds, this is equivalent to worst case ±4.74% change between sites. To put into perspective, previous studies have reported that mean MWF was 16–37% lower in the normal-appearing WM of MS compared to WM of healthy controls (Laule et al., 2004; Faizy et al., 2016; Choi et al., 2018). Flynn et al found that mean MWF was 12% lower in the overall WM in schizophrenia compared to healthy controls (Flynn et al., 2003). Given the small sample size, the magnitude of difference is uncertain so we recommend taking the effect of center or machine into account when doing the analysis in multicenter studies.

While the intersite MWF reproducibility from regional ROI remained high (COV range 4.47–17.89%), frontal brain regions, including genu (17.89%) and minor forceps (12.71%), showed higher variability than others, possibly due to artifact caused by susceptibility, flow and motion. As a comparison, Meyers et al. (2013) reported intersite MWF COV of 15.67 and 17.18% in genu and minor forceps, respectively, using the same MR scanner vendor. The air-filled sinuses produce differences in the tissue magnetic susceptibility resulting in local magnetic field inhomogeneities; it is still unclear how this will affect our quantitative measurements. Furthermore, the genu is located near the frontal horn of the lateral ventricle containing cerebrospinal fluid, which pulsates with the cardiac cycle, which again could introduce artifacts in our measurements.

A power analysis using the G^∗^ Power 3.1 program indicated that based on our pilot study results, for a larger trial, a total sample of 46 people would be needed to detect medium effect size (Cohen’s *d* = 0.49) with 90% power using a paired *t*-test between means with alpha at 0.05. A total sample of 35 people would be needed to achieve 80% power with the same effect size and alpha.

Previous multi-echo T_2_ reproducibility studies using a single vendor scanner have shown good to moderate reproducibility of myelin content measurements (Vavasour et al., 2006; Levesque et al., 2010; Meyers et al., 2013). The data acquired for our study differs from the previous MWF reproducibility studies in several aspects. Our study was performed on 3 T scanners, which improved signal-to-noise in the decay curve by almost 100% compared to 1.5 T (Kolind et al., 2009). The GRASE sequence used in this study (Prasloski et al., 2012b) can be acquired in a clinically feasible time (under 15 min) compared to a single-slice multi-echo spin echo acquisition (1 slice in 26 min) (MacKay et al., 1994). Another advantage of the GRASE acquisition is that data is collected in 3D. This avoids potential magnetization transfer effects between slices that could occur in 2D multi-slice acquisition, which can affect the MWF (Vavasour et al., 2000). To account for B_1_ inhomogeneity, stimulated echo correction was applied to correct for errors in refocusing flip angle (Prasloski et al., 2012a), which will improve the robustness of the analysis. We also studied both regional and global WM. Finally, and most importantly, we studied reproducibility across two scanner vendors located at two sites.

In this study, we were interested in studying the reproducibility of the MWF across two different sites, each using their standard of practice MWI sequence. We believe this best reflects a future multicenter study where the inevitable slight differences between vendors make it impractical to match all sequence parameters as well as hardware. As a consequence of this, different in-plane resolution and imaging acceleration were used at the two sites in this study. Both of these parameters are likely to affect the signal to noise ratio (SNR) of the acquisition. Furthermore, the use of partial k-space acceleration on the Siemens scanner in comparison to parallel imaging on the Philips scanner can also affect the image quality. Another factor that will contribute to differences in SNR between the two sites is the use of different radiofrequency (RF) receiver coils. The Siemens site used a 32-channel coil compared to the 8-channel coil at the Philips site. A larger number of channels in the coil will intrinsically lead to higher SNR and lower g-factor for parallel imaging; however, we would not expect any drastic differences considering that parallel imaging was not used on the Siemens site and acceleration factor of 2 was used on the Philips site (Wiesinger et al., 2005).

Previous studies have investigated the effect of SNR on the MWF through simulations and found that the MWF may be underestimated at low SNR (Bjarnason et al., 2010). In this study, we do not find any results that would support the hypothesis that differences in SNR between the two sites, due to differences in hardware and acquisition, cause significant differences in the MWF. This is an important result, indicating that the MWF estimates are reasonably robust to differences in both hardware and acquisition parameters.

Another limitation of the present study is the small sample size of only 10 participants. Also, scan-rescan data at each site were not collected in this study. However, previous studies that acquired a multi-echo spin echo sequence in brain (mean intrasite MWF COV = 3.99%) (Meyers et al., 2013) and GRASE-derived MWI in spinal cord (Ljungberg et al., 2017) using Philips Achieva 3 T reported good scan-rescan intrasite repeatability, suggesting robustness of the technique. Further, another study that acquired a GRASE-derived MWI in brain (Oh et al., 2014) using a Siemens Magnetom Trio 3 T also reported good scan-rescan intrasite repeatability (mean correlation coefficient = 0.88 ± 0.03).

## Conclusion

This study showed good reproducibility of MWF measurements between two sites with MRI scanners from different vendors. The intersite variability was comparable to previous results using a single MRI scanner vendor (Meyers et al., 2013), without significant bias between sites. Thus, our results support the future use of MWI in studies involving multiple centers and different scanner vendors. Acquiring MWF data across larger groups and populations will allow us to gain deeper insight into pathological processes due to underlying disease progression in demyelinating diseases like MS, which may not be possible with conventional MRI.

## Author Contributions

LL and EL coordinated the study, recruited the participants, collected and analyzed the data, interpreted the results, and drafted the manuscript for intellectual content. DS coordinated the study, collected the data, and revised the manuscript for intellectual content. CF, IV, AR, JC-A, DL, AT, and AM interpreted the results and revised the manuscript for intellectual content. JL designed the study, obtained funding for the data analysis, interpreted the results, and revised the manuscript for intellectual content. SK designed and conceptualized the study, supervised the data analysis, obtained funding for data analysis, interpreted the results, and revised the manuscript for intellectual content.

## Conflict of Interest Statement

The authors declare that the research was conducted in the absence of any commercial or financial relationships that could be construed as a potential conflict of interest.
